# Fatty Acid Profiles and Biological Activities of the Vegetable Oils of *Argania spinosa*, *Pinus halepensis* and *Pistacia atlantica* Grown in Tunisia: A Preliminary Study

**DOI:** 10.3390/molecules29010160

**Published:** 2023-12-27

**Authors:** Marwa Khammassi, Giuseppe Amato, Lucia Caputo, Filomena Nazzaro, Florinda Fratianni, Habiba Kouki, Ismail Amri, Lamia Hamrouni, Vincenzo De Feo

**Affiliations:** 1 Laboratory of Management and Valorization of Forest Resources, National Institute of Research on Rural Engineering, Water, and Forests, Ariana 2080, Tunisia; khammassi_marwa@yahoo.fr (M.K.); amri_amri@live.fr (I.A.); hamrounilam@yahoo.fr (L.H.); 2 Department of Pharmacy, University of Salerno, Via Giovanni Paolo II, 132, 84084 Fisciano, Italy; gamato@unisa.it (G.A.); defeo@unisa.it (V.D.F.); 3 Institute of Food Science, National Research Council, Via Roma, 83100 Avellino, Italy; filomena.nazzaro@isa.cnr.it (F.N.); florinda.fratianni@isa.cnr.it (F.F.); 4 Laboratory of Biotechnology and Nuclear Technology, National Center of Nuclear Science and Technology, Sidi Thabet, Ariana 2020, Tunisia; habibakouki96@gmail.com

**Keywords:** vegetable oils, chemical composition, anti-inflammatory activity, anti-enzymatic activities, antibiofilm activity

## Abstract

Several foods are used in both the nutraceutical and health sectors; vegetable oils, for example, can prevent the onset of numerous diseases. The properties of these oils are related to their chemical composition and primarily to the presence of fatty acids. The present work aimed to determine the chemical profiles of *Argania spinosa*, *Pinus halepensis,* and *Pistacia altantica* oils, used in traditional Tunisian foods, and to evaluate some biological properties. We evaluated their antioxidant, anti-enzymatic, antimicrobial, and anti-inflammatory properties. Linoleic acid was the main component of the three oils*. P. atlantica* oil showed more significant inhibitory activity against the enzymes studied than *A. spinosa* and *P. halepensis*. All three oils showed similar antioxidant and anti-inflammatory activity. Furthermore, *A. spinosa* and *P. halepensis* oils showed antibiofilm activity against *P. aeruginosa*, with 30–40% inhibition. These results focus on the possible use of these oils in the nutraceutical and healthcare sectors.

## 1. Introduction

Recently, interest in plant products for the prevention and treatment of several pathologies has increased. Vegetable oils, for example, are especially used in the pharmaceutical, cosmetic and food industries and could be a good alternative to classic drugs [1]. The biological activities and nutritional values of vegetable oils have been attributed mainly to the presence of fatty acids, even if other compounds like phytosterols are involved in metabolic regulation. In fact, they are capable of inhibiting cholesterol absorption [1,2]. Moreover, polyphenols and terpenoids are notably known for their antioxidant and anti-inflammatory properties [2]. In North Africa, some uncommon species, such as the argan tree [*Argania spinosa* (L.) Skeels, *Sapotaceae*], the Aleppo pine (*Pinus halepensis* Mill., *Pinaceae*), and the Atlas pistachio (*Pistacia atlantica* Desf., *Anacardiaceae*) are used as a source of vegetable oils used in the preparation of dishes, but they also present some vital pharmaceutical properties.

*A. spinosa* is an endemic evergreen tree that grows in southwestern areas of Morocco [3]. The oil from its fruits is rich in fatty acids like oleic and linoleic acids, sterols, tocopherols, phenols, carotenoids, xanthophylls, triterpene alcohols, and squalene. Sour and coworkers reported different activities for argan oil, such as antioxidant, anti-inflammatory, antidiabetic, antihypertensive, antimalarial, anti-hypercholesterolemic, and anti-carcinogenic properties [4].

*P. halepensis* is a species widely distributed in the Mediterranean basin and is used in traditional medicine to treat sexual problems, as an anti-inflammatory, and as an antidiabetic [5]. This oil is rich in monounsaturated and polyunsaturated fatty acids, the main components of linoleic, oleic, and palmitic acids (about 5%) [6,7]. This oil exhibits antioxidant activity and can delay the progression of chronic diseases [8]. *P. atlantica* is widely distributed in North Africa; its seed oil has a pleasant flavor and odor [8]. It mainly contains unsaturated fatty acids such as oleic and linoleic acids [9]. The traditional use of this oil is related to the treatment of several diseases, like stress and stomach diseases, and can act as a tonic, antidiarrheal, and antibacterial [10].

Fatty acids may neutralize free radicals, not allowing their propagation and protecting cells from oxidative damage to DNA [11]. Furthermore, they can act on inflammatory processes, reduce inflammatory cytokines [12], and play an essential role in the physiological functions of the brain. Several studies have shown that alterations in their metabolism are associated with neurological diseases such as Alzheimer’s and Parkinson’s disease [13]. Furthermore, some fatty acids, such as α- and β-unsaturated fatty acids, could inhibit the action of cholinesterases [14]. As well, some fat-soluble constituents were reported for possible anti-α-amylase and anti-α-glucosidase activity, and can be used as a therapeutic food source without many side effects [15]. Considering these findings, the importance of natural substances in the identification and development of antibacterial agents against the emergence of antimicrobial resistance [16], and the fact that cholinesterase inhibitors are the only approved drugs in the treatment of Alzheimer’s disease [17], this research was focused on the fatty acid composition of the oils from *A. spinosa*, *P. halepensis*, and *P. atlantica* and the evaluation of their possible antioxidant, anti-cholinesterase, anti-α-amylase, anti-α-glucosidase, antimicrobial, and anti-inflammatory activities, some of them never studied before for these oils.

## 2. Results

### 2.1. Fatty Acid Profiles

The yields of the oils from *A. spinosa* kernels and *P. atlantica* and *P. halepensis* seeds were 57.2%, 36.9%, and 30.8%, respectively. The fatty acid composition of the oils is reported in Table 1. We recorded high percentages of linoleic acid in all oils. *P. halpensis* oil contained a significant percentage of this acid (66.6%), compared to *A. spinosa* and *P. atlantica* oils (58.9% and 58.1%, respectively). The oil of *P. atlantica* contained the highest percentage of oleic acid (21.2%), followed by *A. spinosa* oil (16.6%). Palmitic acid was the most detected saturated fatty acid in the oils; it was most abundant in *P. atlantica* and *A. spinosa* oils. Linolenic acid was detected in all oils, and *A. spinosa* oil revealed the highest percentage (7.8%). Long-chain unsaturated fatty acids were also detected in oils; arachidic acid was present in all oils, ranging between 1.1 and 4.0%, while gadoleic acid was detected in *A. spinosa* and *P. halepensis* oils, and arachidonic acid was identified only in the oil of *P. halepensis*.

### 2.2. Antioxidant Activity

The antioxidant activity of the oils, evaluated by DPPH, FRAP, and ABTS assays, is reported in Table 2. In the DPPH assay, the effectiveness of the oils was the same, as evidenced by the EC_50_ values. FRAP values varied from 2.46 to 4.93 ± 0.28 mM TE/g; *P. atlantica* showed the highest activity in reducing ferric iron. *P. atlantica* oil showed the highest TEAC value (2.84 mM TE/g) in the ABTS assay.

### 2.3. Activity against Cholinesterases

The inhibitory activity of the oils against AChE and BChE is presented in Table 3. *P. atlantica* oil exhibited higher activity against AChE with an EC_50_ value of 4.81 μg/mL. The EC_50_ value of galantamine used as positive was higher than that of the tested oils. *P. atlantica* oil was also the most active against BchE, with an EC_50_ value of 11.38 μg/mL, lower than galantamine.

### 2.4. Activity against Amylase and Glucosidase

Table 3 reports the inhibitory activity of the oils on α-amylase and α-glucosidase. The three oils showed similar inhibition of α-amylase, with EC_50_ values ranging from 310 to 370 μg/mL. The oil of *P. atlantica* was the most active against α-glucosidase, with an EC_50_ value of 10.23 μg/mL and more active with respect to the acarbose used as a positive control (EC_50_ = 80.13 μg/mL).

### 2.5. Anti-Inflammatory Activity

In the anti-inflammatory test, the three oils were active, with EC_50_ values lower than 2 μg/mL, significantly lower than the control, diclofenac sodium, which showed an EC_50_ of 5.47 μg/mL (Table 3).

### 2.6. Antimicrobial and Antibiofilm Activity

MIC values (Table 4) allowed us to identify the concentration of the oils suitable to be tested to evaluate their potential antibiofilm activity. For this, two tests were carried out: the first on the growth of biofilms and the second on the metabolic pathway of sessile cells present within biofilms.

In the test on mature biofilms, the three oils exhibited quite different behavior from each other (Table 5). The only common feature was their ineffectiveness against *S. aureus*, with inhibition percentages between 0 and 1.29%. However, except for the practically zero activity exerted by the oil of *P. halepensis* vs. *A. baumannii*, the three oils showed inhibitory effects up to 50.30% (oil of *A. spinosa* vs. *A. baumannii*) and in several cases exceeded 40% (45.25 and 42.15% inhibition exerted by *P. halepensis* oil vs. *P. aeruginosa* and vs. *L. monocytogenes*, respectively) and 30% (35.21 and 34.80% inhibition exerted by *P. atlantica* oil vs. *A. baumannii* and *L. monocytogenes*, respectively; 34.54% exhibited by *P. halepensis* oil vs. E. coli; 34.83% shown by *A. spinosa* oil vs. *P. aeruginosa*). In some cases, the inhibitory effect was also observed on the sessile cells’ metabolism within the bacterial biofilm (Table 6). Thus, Phalepensis oil continued to be ineffective against *A. baumannii*.

However, in some cases, no correlation was observed between the results obtained from the previous test and those obtained from the test on the metabolism of sessile cells (Table 6). This was the case, for example, with *E. coli*, against whose metabolism the three oils were utterly ineffective. In this case, the inhibitory activity in the test on the mature biofilm may have been caused by other mechanisms that did not affect the bacterial cellular metabolic pathway and will be the subject of future investigations. Conversely, in the case of *S. aureus*, its metabolism was sensitive to the inhibition exerted by *P. atlantica* oil.

The correlation analysis, considering the most abundant fatty acids (palmitic, oleic, and linoleic acids) and the inhibitory activity of the three oils on the mature bacterial biofilm and sessile cell metabolism, revealed some interesting aspects. Palmitic (r = 0.97) and oleic acids (r = 0.79) potentially influenced the inhibitory effect exerted—more or less consistently—by the three oils against *A. baumannii.* Furthermore, oleic acid seemed to have the most significant influence (r = 0.69) on the oils’ action against the metabolism of the sessile cells of *A. baumannii*. Conversely, the presence of palmitic (r = −0.98) and oleic acids (r = −0.83) seemed to hurt all of the three oils’ inhibition of *E. coli*, against which it seemed instead only the presence of oleic acid was an influence (r = 0.98). The correlation analysis between the fatty acid content and the inhibitory effect that the latter exerted against *L. monocytogenes* permits us to assume that linoleic acid (r = 0.74) influenced the biofilm inhibition of the three oils; on the other hand, oleic acid (r = 0.95) influenced the inhibitory activity of the oils against the metabolism of this bacterium, as opposed to the adverse effect exerted by linoleic acid (r = −0.85). The presence of linoleic acid could be correlated with the inhibition vs. *P. aeruginosa* (r = 0.82), which would at least be able to balance the negative influence exerted by palmitic acid (r = −0.70) but, above all, by oleic acid (r = −0.93). Palmitic acid seemed to harm the inhibitory ability of the three oils vs. *P. aeruginosa*; on the other hand, it would be able to exert an even minimal positive influence on the activity of the three oils on the metabolism of the sessile cells of this pathogen. Finally, regarding *S. aureus*, which was almost entirely resistant to the presence of the three oils, the minimal inhibitory effect could be attributable to linoleic acid. In contrast, oleic (r = 0.65) and palmitic acid (r = 0.29) might contribute to the inhibitory effect of the oils on the metabolism of this bacterium, an effect somewhat “restrained” by the presence of linoleic acid (r = −0.42).

## 3. Discussion

### 3.1. Fatty Acid Profiles

Plant oil content depends on species, environmental conditions, origin, and extraction methods [18]. The yield of *A. spinosa* oil in this study was higher than those reported by Taneva and coworkers [19] (57.2% vs. 40.5%). The yields of *P. halepensis* and *P. atlantica* oils were similar to the data reported in the literature. The *P. halepensis* oil yield from five Tunisian populations depended on the origin, with values varying between 32.92% and 36.85% [6]. Likewise, the yields of *P. atlantica* oil from Algeria and Iran were 39.8 and 26.8%, respectively [20]. Vegetable oils have beneficial effects on human health. They have been used for a long time for the prevention and treatment of many diseases. Their nutritional values have been demonstrated, and their effects have been attributed to their chemical composition, particularly their richness in fatty acids. In this study, linoleic and oleic acids were the main unsaturated fatty acids in *A. spinosa* oil, with percentages of 58.96 and 16.6%, respectively. However, in a Moroccan sample analyzed by Taneva et al. [19] and in a Tunisian oil studied by Hanana et al. [21], oleic acid was present in a significant percentage relative to linoleic acid. Moreover, for saturated fatty acids, the amount of palmitic acid has been similar to that in our sample, but in Hanana’s samples, stearic acid (5.2%) was absent [21].

The *P. halepensis* oil was mainly composed of unsaturated fatty acids, with linoleic acid as the main fatty acid (66.6%). Palmitic, oleic, and linolenic acids were found in percentages of 7.5, 6.7, and 4.5%, respectively. Our data corroborate with those reported in the literature; the study by Khouja et al. on Tunisian samples showed that linoleic acid was the major fatty acid, followed by oleic and palmitic acids [7]. Similar results were found in an Algerian sample, characterized by the presence of linoleic (59.25%), oleic (24.55%), and palmitic acids (4.99%) [22].

The *P. atlantica* oil was rich in linoleic (58.12%), oleic (21.42%), and palmitic acid (14.14%). These results agree with previous reports of linoleic acid as the major unsaturated fatty acid, followed by oleic and palmitic acid [9].

### 3.2. Antioxidant Activity

The results of the DPPH, FRAP, and ABTS assays showed the antioxidant capacities of the three oils. When tested by ABTS and FRAP assays, *P. atlantica* oil exhibbited the main activity. Chelghoum et al. reported similar results for leaves, galls, and fruit oils [23]. Likewise, the literature demonstrated the capacity of *A. spinosa* and *Pinus halpensis* oils to scavenge DPPH radicals. Kamal et al. reported the antiradical capacity of *A. spinosa* seed oil extracted using a hand press (3.46 ± 0.03 mg TE/g dw) [1].

Moreover, the work of Dhibi and coworkers showed that the remaining DPPH radical activity (%) in *P. halapensis* oil was 47.9% after 1 min of incubation [8].

The antioxidant activity of our samples might be attributed to the presence of unsaturated fatty acids, particularly ω-3 fatty acids. Simopoulos’s study reported that α-linolenic acid is a very potent antioxidant and confirmed that vegetable oils are a valuable source of natural antioxidants and can be used in pharmaceutical and food industries [24,25]. Moreover, an α-linolenic acid-enriched diet containing *Camelina sativa* oil (28.8% α-linolenic acid in the diet for two weeks) attenuated oxidative stress in rats [26]. Furthermore, it was demonstrated that α-linolenic acid extracted from linseed oil reduced induced oxidative stress in rats [27]. 

### 3.3. Activity against Cholinesterases

In this study, the oil of *P. atlantica* exhibited the main inhibitory activity against AChE and BChE. No previous studies are available on the action of these three oils on AChE and BChE. The activity shown by *P. atlantica* oil was probably due to the presence of both linoleic and arachidonic acids. Akay and coworkers showed that these fatty acids inhibited AChE with EC_50_ values of 7.95 and 2.78 μM for linoleic and arachidonic acid, respectively [28]. The interest in the interaction of vegetable oils with cholinesterase enzymes has increased over the last few years. Fratianni and colleagues studied the activity of cholinesterases in some seed oils and reported that broccoli and green coffee seed oils showed the best cholinesterase-inhibitory activity [29]. Moreover, the oil from the *Pouteria caimito* Radik. (abiu) seeds exhibited inhibition of AChE (68.40%) and, therefore, is considered a potent inhibitor [30].

### 3.4. Activity against α-Amylase and α-Glusosidase

The three oils were effective against α-amylase, and *P. atlantica* oil was the most active against α-glucosidase. No previous studies on this activity were reported for *P. atlantica* and *P. halepensis* oils. Recently, Daoudi et al. demonstrated the effect of *A. spinosa* oil on the activity of α-glucosidase and α-amylase; the oil exhibits a potent inhibition toward α-amylase, similar to the action of acarbose [31]. This activity may be attributed to the chemical profiles of the oils. It was reported that the liposoluble constituents of the oils could play a crucial role in inhibiting enzymes associated with type 2 diabetes. It was demonstrated that some fatty acids such as oleic, linoleic, and palmitic acids can play a role as α-amylase and α-glucosidase inhibitors [15].

### 3.5. Anti-Inflammatory Activity

The activity of the oils against protein denaturation was studied to evaluate their potential anti-inflammatory activity in vitro. Protein denaturation, and consequently the loss of structure and function, is one of the causes of inflammatory states. Many external stimuli and the action of organic solvents or heat can lead to the denaturation of proteins. So, it is possible to evaluate the anti-inflammatory activity of different substances by assessing their activity in preventing the denaturation of proteins [32]. The three oils showed anti-inflammatory activity, focusing on their possible use in the nutraceutical field. The data obtained agree with those in the literature; in fact, the anti-inflammatory activity of *A. spinosa* oil has already been reported [1]. However, no studies have reported the anti-inflammatory activity of *Pinus halepensis* and *Pistacia atlanica* oil, even if this activity was shown for its extracts [33,34]. This activity is probably due to the high quantity of mono- and polyunsaturated fatty acids, particularly linoleic and oleic acid [35,36].

### 3.6. Antibiofilm Activity

This work studied the potential inhibitory effect of the three oils on the mature biofilm and sessile cell metabolism of five pathogens of food and clinical health interest. No previous literature data were available on this activity. The antibacterial activity of *P. atlantica* oil was confirmed against *P. aeruginosa* and *E. coli*, as observed by Darakhshandeh-Ghahfarokhi et al. [37]. Moreover, *P. atlantica* oil has proved capable of exercising an anti-quorum sensing activity [38]. However, contrary to what was previously observed, *P. atlantica* oil could have been more effective vs. *S. aureus*. The efficacy of *P. atlantica* oil against the mature biofilm of *A. baumannii* confirmed this plant’s usefulness in counteracting emerging, clinically important pathogens and their biofilms [39]. The oil of *P. halepensis* showed good capacity to inhibit the biofilm of different Gram-positive and Gram-negative bacteria [33], also demonstrating good performance in eradicating multispecies oral biofilms [40]. *A. spinosa* oil was the most known among the three oils studied in our work. This oil, for example, inhibited the biofilms of four bacteria (*Streptococcus mutans*, *S. anginosus*, *S. intermedius*, and *Staphylococcus haemolyticus*), and protected against microbial infection [41]. Fratianni et al. ascertained that palmitic, oleic, and linoleic acids could affect the different behaviors of seed oils, and that oleic acid does not always positively influence the inhibitory capacity of some oils against the five strains also used in our experiments [29]. Conversely, in our experiments, the presence of oleic acid seemed to reinforce the inhibitory activity of the oils against *A. baumannii* and negatively affect action against *L. monocytogenes*. This demonstrated once again that fatty acid quali–quantitative composition could affect the biological properties of vegetable oils in a peculiar way, including their effect against mature biofilms [29].

## 4. Materials and Methods

### 4.1. Plant Material

Nuts of *A. spinosa* and seeds of *P. halepensis* and *P. atlantica* were harvested in July 2022 from the botanical gardens of Tunis, Kasserine, and Sidi Bouzid (Tunisia), respectively. The plant material was collected from five trees separated by at least 15 m and mixed to obtain representative samples. Dr. Ismail Amri identified the plant materials, and voucher specimens were deposited in the herbarium section of the Institut National de la Recherche en Génie Rural, Eaux et Forêts (INRGREF), Tunis. Argan nuts were broken to collect the white kernels. Kernels and seeds from the selected species were ground until the obtention of a homogeneous powder suitable for extraction.

### 4.2. Oil Extraction

Ten g of each powder was extracted by a Soxhlet apparatus with n-hexane for six hours. The solvent was evaporated using a rotary evaporator with an operating bath temperature of 60 °C. Samples were placed in amber bottles until further analysis.

### 4.3. Determination of Fatty Acids

Fatty acid methyl esters (FAMEs) were obtained as previously described [29]. The chromatographic separation was performed using an HP-5MS capillary column (30 mm × 0.25 mm, 0.25 μm film thickness) and helium as mobile phase (1 mL/min). FAME injection (1 μL, 10% in CH_2_Cl_2_, *v*/*v*) was carried out in split mode (50:1). The injector temperature was 250 °C, whereas the detector temperatures were 280 and 180 °C for the flame ionization detector (FID) and mass spectrometry (MS), respectively. For the gas chromatography–mass spectrometry (GC–MS) analysis, the ionization voltage, the electron multiplier, and the ion source temperature were set at 70 eV, 900 V, and 230 °C, respectively. The following elution program was used: 220 °C for 6 min increased to 270 °C at 3 °C/min and held at 270 °C for 4 min. The compounds were determined by calculating their Kovats retention index with respect to the standard. The analyses were performed in triplicate, and the values were the mean ± SD of three experiments.

### 4.4. Antioxidant Activity

#### 4.4.1. Extraction

The oils were mixed with acetone (Sigma, Milano, Italy) (1:1, *v*/*v*) and placed for 1 h at 25 °C. Then, a solution of methanol and HCl 1% (1:2, *v*/*v*) was added. The samples were then centrifuged at 13,000 rpm for 5 min; the supernatant was recuperated, and the pellet was treated with the solution of methanol-HCl 1% and centrifuged. The two supernatants were joined and used for the antioxidant tests.

#### 4.4.2. DPPH Test

Free-radical scavenging activity was determined by using the DPPH (1,1-diphenyl-2-picrylhydrazyl radical) assay [29].

The analysis was carried out in microplates by adding 7.5 μL of the sample [previously diluted 1:1, *v*/*v* in DMSO (Sigma, Milano, Italy)] to 303 μL of a 153 mM methanol solution of DPPH. Then, the absorbance of this DPPH solution was measured by a UV-Vis spectrophotometer (Cary Varian, Milano, Italy) as a basis. The percent inhibition of the free radical by DPPH (I%) was calculated with the following formula:

$$I\%=\frac{{A_{sample}-A}_{blank}}{A_{blank}}\times 100$$

where A_blank_ is the absorbance of the control and A_sample_ is the absorbance of the test compound read at 517 nm after 60 min. In addition, the EC_50_ value, referring to the concentration of samples needed to inhibit the activity of 1 mL of DPPH by 50%, was determined.

#### 4.4.3. FRAP Test

Antioxidant power was determined in vitro by the ferric ion reducing antioxidant power assay, following the method of Fratianni et al. [29]. Briefly, ten μL of different sample concentrations was added to 1 mL of FRAP reagent. The antioxidant activity was calculated as the difference in absorbance at 593 nm between the reading at 6 min and the reading at 0 min. These values were related to a standard curve made with different concentrations of Trolox (Sigma Aldrich, Milano, Italy). The results were expressed as the mM of Trolox equivalent/g of oil.

#### 4.4.4. ABTS Test

The ABTS (2,2′-azinobis (3-ethylbenzothiazoline-6-sulfonic acid) diammonium salt) test was performed according to Fratianni et al., using Trolox as standard [29]. Two water solutions of ABTS 7 mM and potassium persulfate 2.45 mM were prepared. The mixture of these two solutions was allowed to stand in the dark at room temperature for 16 h before use to form the ABTS radical (ABTS•+. The ABTS radical solution was diluted with distilled water to obtain an absorbance of 1.00 at 734 nm. The samples (final concentrations 0.0001–0.01 mg/mL) or Trolox standards (final concentration 0–20 mM) were added to the diluted ABTS•+ solution; the absorbance reading was read after 6 min (Cary Varian, Milano, Italy). The results were expressed as mM Trolox equivalent antioxidant capacity (TEAC) of samples.

### 4.5. Activity against Cholinesterases

The spectrophotometric method of Khedri et al. was used to evaluate the in vitro inhibitory activity on acetylcholinesterase (AChE) and butyrylcholinesterase (BChE) [42]. Briefly, in a total volume of 1 mL, 415 μL of Tris-HCl buffer 0.1 M (pH 8), 10 μL of a DMSO solution of the oils at different concentrations, and 25 μL of a solution containing 0.28 U/mL of AChE (or BChE) were placed for 15 min at 37 °C. Then, we added a water solution of acetyltiocholine iodide (or butyrylthiocholine iodide) 1.83 mM (75 μL) and 475 μL of 5,5′-dithiobis(2-nitrobenzoic acid). The mixture was placed for 30 min at 37 °C. The absorbance was read at 405 nm in a spectrophotometer (Thermo Scientific Multiskan GO, Monza, Italy). Galantamine was the positive control. The AChE and BChE in vitro inhibitory activities were expressed in terms of the EC_50_ value (μg/mL required to inhibit 50% of enzyme activity).

### 4.6. Activity against α-Amylase and α-Glucosidase

#### 4.6.1. α-Amylase Inhibition Assay

Amylase activity was measured using Bernfeld’s method, with slight modifications [42,43]. One hundred microliters of different concentrations of vegetable oils (obtained by dissolving oils in DMSO 1%) was added to 200 μL of 20 mM sodium phosphate buffer (pH = 6.9) and 100 μL amylase water solution (10 U/mL). Then, the mix was placed at 37 °C for 10 min. After, 180 μL of 1% starch solution was added and set at 37 °C for 20 min. One hundred and eighty microliters of 3,5 dinitrosalycyclic acid water solution (96 mM) was added to the mixture and placed in a block heater at 100 °C for 10 min. Then, the solution was diluted with 600 μL of distilled water. The absorbance of the solution was read at 540 nm in a UV spectrophotometer (Thermo Fischer Scientific, Vantaa, Finland).

#### 4.6.2. α-Glucosidase Inhibition Assay

α-Glucosidase inhibitory activity was evaluated as reported by Si et al., with some modifications [42,44].

Briefly, the assay was performed in 96 multiwell plates; 150 μL of 0.1 M phosphate buffer at pH 7.0 was added to each well; successively, ten μL of vegetable oils dissolved in DMSO to obtain different concentrations was added. Then, 15 μL of the α-glucosidase enzyme water solution (1 U/mL) was added to each well, and the plate was incubated at 37 °C; after 5 min, 75 μL of the substrate (2.0 mM) 4-nitrophenyl α-D-glucopyranoside was added, and successively the plate was placed for 10 min at 37 °C. The absorbance was measured at 405 nm in a UV spectrophotometer (Thermo Fischer Scientific, Vantaa, Finland). Acarbose was the positive control, whereas the phosphate buffer in place of the sample was used as a negative control. Inhibition of the enzyme was calculated, and the results were expressed as EC_50_ values.

The following formula calculated the percent inhibition of activity for cholinesterases, α-amylase, and α-glucosidase:

$$\%=\frac{A_{0}-A_{1}}{A_{0}}\times 100$$

where A_0_ is the absorbance of the negative control (enzyme and substrate without any inhibitor) and A_1_ is the absorbance of the sample. The concentration providing 50% inhibition (EC_50_) was obtained by plotting the inhibition percentage against sample concentrations.

### 4.7. Anti-Inflammatory Activity

Anti-inflammatory activity was evaluated by measuring the inhibition of BSA (bovine serum albumin denaturation) [29]. The reaction mixture (5 mL) comprised 0.2 mL BSA solution 0.5% (*w*/*v*) (96% purity, Sigma, Milano, Italy) in 0.05 M Tris–phosphate-buffered saline solution, pH 6.5, 2.8 mL of Tris–phosphate-buffered saline solution, and 2 mL of varying concentrations of samples. One mL of BSA containing methanol was the control. After heating at 72 °C and the subsequent cooling, the absorbance was calculated at 660 nm using a Thermo Scientific Multiskan GO spectrophotometer (Thermo Fischer Scientific, Vantaa, Finland). Diclofenac sodium represented the reference drug.

### 4.8. Antibacterial Activity

#### 4.8.1. Microorganisms and Culture Conditions

The bacterial strains *Acinetobacter baumannii* (ATCC 19606), *Escherichia coli* (DSM 8579), *Pseudomonas aeruginosa* (DSM 50071), *Listeria monocytogenes* (ATCC 7644), and *Staphylococcus aureus* subsp. *aureus* Rosebach (ATCC 25923), purchased from Deutsche Sammlung von Mikroorganismen und Zellkulturen (Braunschweig, Germany), were used in the experiments. Before the antimicrobial assays, they were cultured in Luria broth (LB, Sigma Aldrich Italia, Milano, Italy) for 18 h at 37 °C (*A. baumannii* was grown at 35 °C) and 80 rpm (Corning LSE, Pisa, Italy).

#### 4.8.2. Minimal Inhibitory Concentration (MIC)

The resazurin microtiter plate assay determined the MIC [29]. The tests were performed in flat-bottomed 96-well microtiter plates placed at 37 or 35 °C (depending on the strain) for 24 h. The MIC value was identified by the color change from dark purple to colorless. Sterile DMSO and tetracycline (dissolved in DMSO, 1 mg/mL) were used as negative and positive controls, respectively. Experiments were carried out in triplicate; the results were expressed as the mean ± standard deviation.

#### 4.8.3. Inhibition of Mature Biofilm

The capacity of the oils to affect a mature bacterial biofilm was assessed in flat-bottomed 96-well microtiter plates (Falcon, VWR International, Milano, Italy) [29]. The overnight bacterial cultures were adjusted to 0.5 McFarland with fresh culture broth. Afterward, 10 μL of the bacterial cultures was added to each well, and 240 μL sterile Luria–Bertani broth (Sigma Aldrich Italia, Milano, Italy) was added. After 24 h, the culture broth was eliminated, and 10 μL/mL and 20 μL/mL of each oil were added, and with sterile Luria–Bertani broth, a final volume of 250 μL was reached. The plates were closed with parafilm tape to avoid the evaporation of material in the wells and incubated for 24 h at 37 or 35 °C. After the elimination of the planktonic cells, sessile cells were washed twice with sterile phosphate-buffered saline (PBS). Then, the plates were placed for 10 min under a laminar flow hood before adding 200 μL of methanol in each well for 15 min to allow the fixation of the sessile cells. Methanol was removed, and each plate was left to dry; then, 200 μL of crystal violet solution 2% *w*/*v* was added to each well. After 20 min, the staining solution was removed and the plates were washed with sterile PBS and left to dry. The bound dye was released by adding 200 μL of glacial acetic acid (20% *w*/*v*) to each well. The absorbance was measured at 540 nm (Cary50Bio, Varian). The inhibitory action on the mature biofilm (expressed as percent value) was calculated with respect to the control (represented by the bacterial cells grown without the presence of the samples, where inhibition was considered 0%).

#### 4.8.4. Inhibition of Bacterial Metabolism

The effects of the oils on the metabolic activity of the sessile cells within the bacterial biofilm after 24 h were also evaluated through the 3-(4,5-dimethylthiazol-2-yl)-2,5-diphenyltetrazolium bromide (MTT) colorimetric method [29]. After another 24 h of incubation, the culture broth was eliminated; then, 150 μL of PBS and 30 μL of 0.3% of MTT (Sigma, Milano, Italy) were added, keeping the microplates at 37 or 35 °C (depending on the strain). After two hours of incubation, the MTT solution was removed, and each well was washed with a sterile physiological solution. Two hundred microliters of dimethyl-sulfoxide (DMSO) were added to the dissolution of the formazan crystals, which were read at 570 nm (Cary50Bio, Varian) after two hours at 37 or 35 °C (depending on the strain).

### 4.9. Statistical Analysis

The tests were performed in triplicate, and the results were expressed as the mean ± SD. The results were analyzed using GraphPad Prism 6.0 software (GraphPad Software Inc., San Diego, CA, USA) with two-way ANOVA followed by Dunnett’s multiple comparison test or one-way ANOVA followed by Tukey’s post hoc test. The differences between individual means were considered significant at (*p* < 0.05).

## 5. Conclusions

The presence of mono- and polyunsaturated fatty acids highlighted the use of these vegetable oils in the nutraceutical and health fields. These oils could represent a natural resource against the onset of infections caused by some pathogens; in particular, *A. spinosa* oil was active against mature *A. baumanii* biofilm and *P. halepensis* oil inhibited *E. coli* and *L. monocytogenes* biofilm growth. The metabolism of *A. baumanii* and *S. aureus* bacterial sessile cells in mature biofilm was inhibited by *P. atlantica* oil; the metabolism of *P. aeruginosa* was inhibited by *A. spinosa* oil; all tested oils inhibited *L. monocytogenes*. Furthermore, the anti-inflammatory activity of all three oils suggests their possible use as natural anti-inflammatories through their presence in a dietary plan. However, the use of these oils would not be limited to the nutraceutical sector alone; in fact, the reported *P. atlantica* oil anticholinesterase activity may suggest its possible use in preventing neurodegenerative diseases. Furthermore, the *P. atlantica* oil was also the most active toward α-amylase and α-glucosidase enzymes, which could also be used to avoid the onset of pathologies linked to diabetes.

## Figures and Tables

**Table 1:** Fatty acid composition (%) of *Argania spinosa*, *Pistacia atlantica,* and *Pinus halepensis* oils.

		%		
RT		*A. spinosa*	*P. halepensis*	*P. atlantica*
24.0	Myristic acid (C14:0)	0.0 ± 0.0 ^a^	0.0 ± 0.0 ^a^	2.0 ± 0.5 ^b^
24.4	Palmitic acid (C16:0)	14.9 ± 0.8 ^a^	7.5 ± 0.5 ^b^	14.1 ± 0.6 ^a^
25.8	Stearic acid (C18:0)	0.0 ± 0.0 ^a^	0.1 ± 0.04 ^b^	0.0 ± 0.0 ^a^
27.6	Oleic acid (C18:1)	16.6 ± 0.7 ^b^	6.7 ± 0.5 ^a^	21.4 ± 0.9 ^c^
27.8	Linoleic acid (C18:2)	58.9 ± 1.3 ^a^	66.6 ± 2.6 ^b^	58.1 ± 1.9 ^a^
28.2	Linolenic acid (C18:3)	7.8 ± 0.6 ^c^	4.5 ± 0.3 ^b^	2.7 ± 0.4 ^a^
31.2	Arachidic acid (C20:0)	1.3 ± 0.1 ^a^	3.3 ± 0.9 ^b^	1.1 ± 0.4 ^a^
31.6	Gadoleic acid (C20:1)	1.8 ± 0.6 ^b^	2.3 ± 0.2 ^b^	0.0 ± 0.0 ^a^
31.8	Arachidonic acid (C20:4)	0.0 ± 0.0 ^a^	4.0 ± 0.8 ^b^	0.0 ± 0.0 ^a^

Different letters indicate mean values significantly different at *p* < 0.05, according to a one-way ANOVA followed by Tukey’s post hoc test.

**Table 2:** Antioxidant activity of the oils evaluated by DPPH, ABTS, and FRAP assays.

	DPPH	FRAP	ABTS
	EC_50_	mM Trolox Eq/g of Oils	mM Trolox Eq/g of Oil
*A. spinosa*	0.36 ± 0.05 ^a^	2.46 ± 0.12 ^a^	1.88 ± 0.02 ^a^
*P. halepensis*	0.35 ± 0.07 ^a^	2.84 ± 0.14 ^a^	2.28 ± 0.02 ^b^
*P. atlantica*	0.35 ± 0.06 ^a^	4.93 ± 0.28 ^b^	2.84 ± 0.07 ^c^

Data are the means ± standard deviation of three experiments. Different letters indicate mean values significantly different at *p* < 0.05, according to a one-way ANOVA followed by Tukey’s post hoc test.

**Table 3:** Anti-enzymatic and anti-inflammatory activity of vegetable oils.

	Anti-Enzymatic Activity				Anti-Inflammatory Activity
	EC_50_ μg/mL				EC_50_ μg/mL
	AChE	BChE	α-Amylase	α-Glucosidase	
*A. spinosa*	12.97 ± 0.09 ^c^	26.36 ± 2.38 ^c^	310.21 ± 30.03 ^c^	1310.97 ± 8.62 ^d^	1.23 ^c^ ± 0.75
*P. halepensis*	na	na	350.14 ± 10.13 ^c^	na	1.86 ^c^ ± 0.22
*P. atlantica*	4.82 ± 0.48 ^d^	11.38 ± 1.98	370.22 ± 10.31 ^c^	10.23 ± 1.56 ^d^	1.51 ^d^ ± 0.02
Galantamine	15.21 ± 0.15	14.51 ± 0.27	/	/	/
Acarbose	/	/	4.48 ± 0.01	80.13 ± 0.95	/
Diclofenac	/	/	/	/	5.47 ± 0.32

Data are the means ± standard deviation of three experiments. ^c^ *p* < 0.001, ^d^ *p* < 0.0001 vs. control (a one-way ANOVA followed by Dunnett’s multiple comparison test). na: not active.

**Table 4:** MIC (μL/mL) of the oils.

	AB	EC	LM	PA	SA
*A. spinosa*	25 ^ns^ ± 2	25 ^ns^ ± 2	28 ^ns^ ± 2	27 ^ns^ ± 3	>30 ^a^
*P. halepensis*	26 ^ns^ ± 3	24 ^ns^ ± 2	28 ^ns^ ± 1	26 ^ns^ ± 2	>30 ^a^
*P. atlantica*	25 ^ns^ ± 2	27 ^ns^ ± 1	27 ^ns^± 3	27 ^ns^ ± 2	>30 ^a^
Tetracycline	25 ± 2	25 ± 2	28 ± 1	28 ± 1	28 ± 1

Results are the mean of three experiments ± SD. AB: *A. baumannii*; EC: *E. coli*; LM: *L. monocytogenes*; PA: *P. aeruginosa*; SA: *S. aureus*. ns: not significantly different vs. tetracycline*;* ^a^ *p* < 0.5; compared with the average values of control (ANOVA followed by Dunnett’s multiple comparison test).

**Table 5:** Inhibitory activity of vegetable oils on the mature biofilm.

CV (24 h)	*P. halepensis* 10 μL/mL	*P. halepensis* 20 μL/mL	*A. spinosa* 10 μL/mL	*A. spinosa* 20 μL/mL	*P. atlantica* 10 μL/mL	*P. atlantica* 20 μL/mL
*A. baumannii*	0.00 ± 0.00	3.31 ^a^ ± 0.44	19.03 ^a^ ± 1.21	50.30 ^c^ ± 3.67	0.00 ± 0.00	35.21 ^b^ ± 2.55
*E. coli*	17.26 ^a^ ± 1.04	34.54 ^b^ ± 3.67	0.00 ± 0.00	15.44 ^a^ ± 1.98	0.00 ± 0.00	20.59 ^a^ ± 1.67
*L. monocytogenes*	15.65 ^a^ ± 1.15	42.15 ^c^ ± 1.05	14.70 ^a^ ± 1.02	15.68 ^a^ ± 2.05	14.34 ^a^ ± 0.57	34.80 ^b^ ± 2.67
*P. aeruginosa*	22.13 ^b^ ± 1.67	45.25 ^c^ ± 2.42	26.35 ^b^ ± 1.02	34.83 ^b^ ± 2.44	13.90 ^a^ ± 0.98	15.55 ^a^ ± 1.05
*S. aureus*	0.00 ± 0.00	1.29 ± 0.08	0.00 ± 0.00	0.00 ± 0.00	0.000.00	1.09 ± 0.11

Tests were performed using 10 and 20 μL/mL of sample. All tests were performed in triplicate. Results are expressed as percentages (mean ± SD) and calculated assuming the control (untreated bacteria = zero). a: *p* < 0.1; b: *p* < 0.01; c: *p* < 0.001, compared with the control (ANOVA followed by Dunnett’s multiple comparison test).

**Table 6:** Inhibitory activity of the oils on the metabolism of the bacterial sessile cells in mature biofilms.

MTT (24 h)	*P. halepensis* 10 μL/mL	*P. halepensis* 20 μL/mL	*A. spinosa* 10 μL/mL	*A. spinosa* 20 μL/mL	*P. atlantica* 10 μL/mL	*P. atlantica* 20 μL/mL
*A. baumannii*	0.00 ± 0.00	4.20 ^a^ ± 0.41	0.00 ± 0.00	0.00 ± 0.00	32.01 ^b^ ± 2.55	49.30 ^c^ ± 4.02
*E. coli*	0.00 ± 0.00	0.00 ± 0.00	0.00 ± 0.00	0.00 ± 0.00	0.00 ± 0.00	0.00 ± 0.00
*L. monocytogenes*	27.79 ^b^ ± 1.67	32.05 ^b^ ± 1.12	26.31 ^b^ ± 2.02	33.83 ^b^ ± 3.15	31.17 ^b^ ± 2.91	36.52 ^b^ ± 2.67
*P. aeruginosa*	0.00 ± 0.00	23.56 ^b^ ± 2.48	0.00 ± 0.00	32.09 ^b^ ± 4.01	12.45 ^a^ ± 1.12	19.85 ^a^ ± 1.56
*S. aureus*	0.00 ± 0.00	2.47 ^a^ ± 0.28	0.00 ± 0.00	0.00 ± 0.00	0.000.00	17.26 ^a^ ± 1.92

Tests were performed using 10 and 20 μL/mL of sample. All tests were performed in triplicate. Results are expressed as percentages (mean ± SD) and calculated assuming the control (untreated bacteria = zero). a: *p* < 0.1; b: *p* < 0.01; c: *p* < 0.001, compared with the control (ANOVA followed by Dunnett’s multiple comparison test).

## Data Availability

Data are contained within the article.
